# How Employee–AI Collaboration Influences Coworkers’ Helping Behaviour: An Attribution Theory Perspective

**DOI:** 10.3390/bs16060985

**Published:** 2026-06-12

**Authors:** Yepeng Wu, Yuanyuan Jiao

**Affiliations:** 1 School of Economics and Management, Hubei University of Technology, Wuhan 430068, China; 2 Hubei Digital Industrial Economy Development Center, Hubei University of Technology, Wuhan 430068, China; 3 Business School, Nankai University, Tianjin 300071, China; 1120170897@mail.nankai.edu.cn

**Keywords:** employee–AI collaboration, laziness attribution, responsibility-avoidance attribution, coworker helping behaviour, human–AI task interdependence, attribution theory

## Abstract

As artificial intelligence (AI) is increasingly integrated into the workplace, employee–AI collaboration is evolving from a personal productivity tool to a social cue that coworkers can observe and interpret. Existing research has largely emphasised the performance and well-being effects of employee–AI collaboration; however, few studies have revealed, from the observer’s perspective, its potential negative spillover mechanisms on coworkers’ helping behaviour. Based on attribution theory, this study constructs a theoretical model of ‘employee–AI collaboration–coworker attributions–coworker helping behaviour’, distinguishing two mechanisms—laziness attribution and responsibility-avoidance attribution—and examines the boundary role of human–AI task interdependence. Study 1, based on 375 two-wave coworker survey responses, tested the hypotheses using hierarchical regression and bootstrapping methods. Study 2 employed a 2 × 2 scenario experiment to further test the effects of employee–AI collaboration and human–AI task interdependence on coworker attributions and willingness to help. The results indicate that higher perceived employee–AI collaboration is associated with lower coworker helping behaviour; laziness attribution and responsibility-avoidance attribution play a mediating role between perceived employee–AI collaboration and coworker helping behaviour. The higher the human–AI task interdependence, the more likely coworkers are to interpret employee–AI collaboration as laziness or responsibility-avoidance, thereby reinforcing the aforementioned negative effects.

## 1. Introduction

Artificial intelligence (AI), exemplified by generative AI, robots, and intelligent decision systems, is reshaping organisational operations and patterns of workplace interaction. Compared with traditional information technologies, AI is no longer a passive tool for executing instructions; it exhibits greater autonomy and interactivity in complex cognitive tasks, including information generation, pattern recognition, solution recommendations, and decision support (Chowdhury et al., 2023; Tan & Li, 2025). In contexts such as medical diagnosis, customer service, and process decision-making, employees increasingly exchange information with AI, jointly diagnose problems, and make decisions, thereby making employee–AI collaboration increasingly prevalent. However, while employee–AI collaboration may enhance employee productivity and organisational efficiency, it may also lead to a “social evaluation penalty” from coworkers: observers may perceive individuals who use AI to complete tasks as having exerted less effort, thereby giving them lower ratings for competence and motivation (Reif et al., 2025). A recent survey showed that fear of being perceived as lazy is one of the primary concerns employees have regarding the use of AI (Asana, 2024). Claessens et al. (2025) also indicated that outsourcing tasks to AI, even with enhanced efficiency, may weaken the evaluation of users’ authenticity, care, and moral qualities. These findings suggest that even in organisations that encourage AI use, coworkers may view employee–AI collaboration negatively, potentially reducing their helping behaviour.

Latest studies have begun examining the interpersonal consequences of AI use. Reif et al. (2025) documented the social-evaluative penalties associated with AI collaboration. Tissot and Roth (2026) demonstrated that AI use lowers moral evaluations because of perceptions of lower effort. Zhou et al. (2025) found that coworkers’ laziness attributions and moral perception of employees’ AI use influence helping behaviour. Although these studies provide an important foundation for understanding the social evaluative consequences of employee–AI collaboration, three gaps remain. First, existing research has focused primarily on the general evaluative penalties imposed by observers on AI users, with less investigation into how coworkers within an organisation adjust their helping behaviour in AI collaboration contexts (Reif et al., 2025). Second, while Zhou et al. (2025) interpreted the negative impact mechanisms of AI use through the lens of moral perception, this work has not yet revealed the antecedent mechanisms leading to such perceptions, which limits the development of actionable managerial interventions for addressing the social penalties associated with AI. Third, current studies have mostly focused on the moderating effects of individual employee characteristics (N. Jia et al., 2024) or organisational features (Man Tang et al., 2022) on employee–AI collaboration outcomes, relatively overlooking the critical boundary condition of employee–AI task interdependence.

Attribution theory provides a suitable perspective for explaining the interpersonal consequences of employee–AI collaboration. It posits that observers infer actors’ motives from behavioural cues, forming attitudes and behavioural responses (Harvey et al., 2014). In this study, perceived employee–AI collaboration constitutes a hybrid behavioural cue: coworkers can observe an employee’s use of AI and task outputs but cannot fully perceive covert labour, such as prompt design, information filtering, output verification, and judgement correction (Hagtvedt, 2025). Thus, perceived employee–AI collaboration is not merely a technological choice; it may also be viewed as a socially evaluable behavioural cue. This study does not assume that perceived employee–AI collaboration necessarily leads to negative evaluations. It examines how negative attributions are activated when effort processes are opaque, and contribution sources and responsibility boundaries are unclear. On the one hand, employee–AI collaboration may reduce the visibility of an employee’s effort process to their coworkers (Raj et al., 2026), leading coworkers to interpret it as a strategy for reducing effort, taking shortcuts, or over-relying on technology, thereby forming attributions of laziness. On the other hand, employee–AI collaboration may also alter the responsibility structure of work outcomes. When task results are co-generated by employees and AI, coworkers may find it difficult to judge whether the outcome stems from the employee’s judgement or the AI’s output (McNealis, 2026), leading to concerns that employees might use AI to blur the boundaries of responsibility and weaken or shift accountability, thereby forming responsibility-avoidance attribution. Furthermore, whether coworkers view employee–AI collaboration as a legitimate tool use or as shirking and blame-shifting depends strongly on the degree of human–AI task interdependence. Human–AI task interdependence refers to the extent to which employee and AI tasks are mutually dependent, connected, and influential in workflows, inputs, outputs, and outcome formation (Tan & Li, 2025). When human–AI task interdependence is high, employee judgement, AI output, and subsequent task progression are tightly coupled, making it difficult for coworkers to discern the boundary between individual effort and AI contribution and to assign responsibility when outcomes are poor. Consequently, laziness and responsibility-avoidance attributions become more likely. However, existing research often treats perceived employee–AI collaboration as a homogeneous behaviour (Teng et al., 2023), overlooking task characteristics at the work-design level in human–AI hybrid teams. As task boundaries between employees and AI vary significantly across work contexts, how task interdependence shapes coworkers’ attributional inferences remains unexplored.

Therefore, this study adopts the perspective of coworker observers and employs attribution theory to examine whether and how perceived employee–AI collaboration affects helping behaviour. First, it investigates whether perceived employee–AI collaboration reduces coworker helping behaviour. Second, it examines the mediating roles of laziness and responsibility-avoidance attribution in the relationship between perceived employee–AI collaboration and coworker helping behaviour. Finally, it tests the moderating effect of human–AI task interdependence on the relationship between perceived employee–AI collaboration and these two attributional mechanisms.

This study makes three main contributions. First, although existing research has examined the social evaluative penalties faced by AI users, it has primarily focused on the overall impressions of general observers of such users (Claessens et al., 2025; Reif et al., 2025). This study extends the literature on social-evaluative penalties from general evaluation contexts to coworker interactions within organisations, revealing the negative spillover effects of perceived employee–AI collaboration on helping behaviour. Second, Zhou et al. (2025) found that AI usage influences coworker helping behaviour through perceived morality; however, the antecedent mechanisms underlying these moral perceptions remain unknown. This study further identifies two antecedent attribution mechanisms for perceived morality–laziness attribution and responsibility-avoidance attribution, and compared to laziness attribution, responsibility-avoidance attribution serves as the dominant path, introducing the responsibility attribution mechanism into the human–AI hybrid workplace context (Sahebi et al., 2026). Third, while the traditional literature on task interdependence has primarily focused on all human collaboration contexts (Chugh et al., 2014), this study extends the concept of task interdependence from traditional all-human collaboration contexts to employee–AI collaboration contexts and further reveals the asymmetric moderation of human–AI task interdependence; when task interdependence is high, coworkers are more likely to interpret it as laziness and responsibility-avoidance.

## 2. Literature Review

### 2.1. Perceived Employee–AI Collaboration

Employee–AI collaboration refers to the interactive behaviour where employees treat AI as a collaborator in their work, engaging in activities such as information exchange, joint diagnosis, and collective decision-making (Kong et al., 2023). Unlike automation or traditional computing, AI has evolved from a passive tool into an active agent capable of autonomous decision-making under uncertainty, even influencing employees’ subsequent judgements and actions during stages such as information filtering, content revision, and final integration (Tang et al., 2023). It should be noted that because the key stages of employee–AI collaboration—such as prompt design, information filtering, result verification, and manual correction—are often not fully visible to coworkers, coworkers typically judge the degree of collaboration between a target employee and AI based on observable cues (C. Xu et al., 2026). Therefore, following Zhou et al. (2025), this study focuses on perceived employee–AI collaboration. Human–AI task interdependence refers to the extent to which employee and AI tasks are mutually dependent and influence workflows, inputs, and outputs, as well as the formation of outcomes. It reflects a characteristic of the task structure rather than the frequency, intensity, or degree of psychological dependence of employees’ AI usage. Thus, employee–AI collaboration describes a behavioural process at the employee level, whereas human–AI task interdependence describes structural characteristics at the task level. Consequently, perceived employee–AI collaboration does not necessarily imply high human–AI task interdependence. Most existing research on AI collaboration focuses on its impact on employees (see Table 1). One stream emphasises the positive effects of AI collaboration, arguing that AI can improve employees’ information-processing efficiency, task performance, creativity, and service quality (Bai et al., 2025; Doshi & Hauser, 2024; Shaikh et al., 2023). Another stream examines potential negative consequences, including technostress, algorithm aversion, occupational monitoring, work alienation, and counterproductive behaviours (Cao et al., 2023; Hai et al., 2025; Zhu et al., 2026). These studies contribute to understanding how AI collaboration affects the users themselves, but they relatively overlook the interpersonal evaluative consequences that may arise once AI collaboration is embedded within organisational social relationships. The latest research has begun to shift the focus of evaluation from “AI systems” to “AI users,” exploring the impression management of AI collaborators by third-party observers. Employee–AI collaboration not only affects the users themselves but also alters others’ social evaluations of them. In contexts such as healthcare, law, recruitment, and content production, when professionals or job seekers are perceived to rely on algorithms or generative AI, evaluators tend to lower their ratings of competence, effort, authenticity, and trustworthiness (Jungherr & Rauchfleisch, 2025; Reif et al., 2025). Leszczyński et al. (2026) found that even when controlling for whether job application materials were actually generated by AI, as long as recruiters perceived that the applicant used AI, the applicant still faced a significant hiring penalty; applicants perceived as highly likely to have used AI received overall evaluations 40.6% lower than those perceived as not having used AI. This finding suggests that the AI-use penalty does not necessarily stem from AI actually reducing task quality, but may arise from observers’ subjective perceptions and social inferences regarding AI usage. Claessens et al. (2025) also pointed out that even when AI is used as a collaborative tool rather than a complete replacement for human labour, observers may still perceive the user as lazier and their work or actions as lacking authenticity and care. Zhou et al. (2025) extended this perspective to the context of workplace peer assistance, finding that coworkers’ negative evaluations of an employee’s AI use affect their willingness to help. These studies indicate that employee–AI collaboration may trigger a penalty mechanism that shifts from “technical evaluation” to “interpersonal evaluation.” While the aforementioned studies provide an important foundation for understanding the interpersonal consequences of employee–AI collaboration, there remains room for further expansion. First, research on algorithm aversion primarily focuses on whether individuals adopt or reject AI outputs, or evaluators’ judgements regarding the competence, morality, and authenticity of AI users, with less investigation into how these evaluations translate into specific interpersonal behaviours within organisations. Second, existing research on the penalties of AI use has mostly focused on outcomes such as competence evaluations and moral perceptions (Kırcaburun & Özdemir, 2026), yet it has not revealed in a fine-grained manner how the antecedent mechanisms of moral perception formation influence helping behaviour. Finally, employee–AI collaboration is not a homogeneous behaviour; its interpersonal consequences may depend on the interdependence between the employee and AI within the task process. Therefore, this study explores the impact of employee–AI collaboration on coworker helping behaviour from the perspective of coworker observers.

### 2.2. Attribution Theory

Attribution theory, proposed by Heider (1958), posits that people seek to understand and predict the social environment by inferring the causes of others’ behaviour (Harvey et al., 2014). Observers infer the reasons and motives behind others’ actions from the available cues and adjust their behavioural responses accordingly (Malle, 2006). In recent years, attribution theory has been widely applied in organisational behaviour and management research to explain, from a third-party perspective, how coworkers interpret and respond to employee behaviour, including job crafting (Xiao et al., 2023), organisational citizenship behaviour (Harvey et al., 2014), recruitment and algorithmic evaluation behaviour (Schilke & Reimann, 2025), and helping behaviour (Zhou et al., 2025). Attribution theory also emphasises that the same behavioural cue does not necessarily lead to a unidirectional attributional judgement; observers interpret the meaning of a behaviour by integrating situational information, normative cues, and responsibility cues. Therefore, employee–AI collaboration does not inevitably trigger negative attributions from coworkers; when AI collaboration is explicitly authorised, widely accepted by the team, and has clear responsibility boundaries, it may be interpreted as a reasonable task requirement or an organisational norm. However, in a workplace setting, coworkers often find it difficult to fully grasp a target employee’s task process, true motivations, and hidden constraints; therefore, it is precisely this incompleteness of information from an observer’s perspective that prompts them to rely on observable behavioural cues to infer effort and accountability, adjusting their helping behaviour (Rodell & Lynch, 2016). In general, any form of external assistance weakens the diagnosticity of task outcomes for attributing contributions to individual actors. When employees complete tasks independently, coworkers are more likely to attribute task outcomes to the employee’s effort, ability, and conscientiousness; however, when employees complete tasks with AI, work outcomes reflect both employee input and AI support, making contribution boundaries more ambiguous (Hohenstein & Jung, 2020).

Compared to general tools, AI possesses strong agency, generativity, and quasi-agentic qualities, even proactively delegating tasks to human employees, which provides a highly ambiguous behavioural cue for peer attribution (Zhou et al., 2025). In coworker-help situations, coworkers not only care whether the target employee was efficient but also whether they exerted appropriate effort and assumed corresponding responsibility. Because AI has strong automation, generative, and decision-support capabilities, it can substitute for some cognitive and judgemental work that the employee would otherwise have performed personally; coworkers may engage in counterfactual reasoning, imagining whether the employee would have exerted equal effort or borne the same responsibility without AI assistance. Consequently, employee–AI collaboration may reduce coworkers’ certainty about the employee’s personal effort and assumption of responsibility. First, the “labour-saving” attribute of AI may reinforce coworkers’ scepticism regarding employees’ efforts. When it is difficult for coworkers to observe an employee’s implicit investment in stages such as screening, verification, and integration, they are more likely to interpret employee–AI collaboration as a strategic choice to reduce effort, rely on technology, or take shortcuts, thereby forming a laziness attribution (Reif et al., 2025). Second, the quasi-agency of AI and the characteristics of human–AI co-production may alter the responsibility structure of work outcomes (Wang et al., 2023). When task results are jointly generated by employees and AI, coworkers may find it difficult to judge whether the outcome stems from the employee’s judgement or the AI’s output. Consequently, they may worry that employees are using AI to blur responsibility boundaries, weaken individual accountability, or shift blame, leading to responsibility-avoidance attribution (McNealis, 2026; L. Xu et al., 2026). The aforementioned attribution mechanisms have significant explanatory power regarding coworkers’ helping behaviour. Helping behaviour is a voluntary, resource-consuming, extra-role behaviour that may involve responsibility implications (H. Jia et al., 2021). Whether coworkers provide help depends not only on whether the target employee is efficient or innovative but also on whether they are perceived as hardworking and conscientious. Laziness attribution occurs at the level of task input and process evaluation, emphasising the judgement of reciprocity in helping; responsibility-avoidance attribution primarily occurs at the level of task outcomes and responsibility evaluation, emphasising the riskiness of helping. Therefore, based on attribution theory, this study explores the mediating roles of laziness attribution and responsibility-avoidance attribution between employee–AI collaboration and coworkers’ helping behaviour.

## 3. Research Hypotheses

### 3.1. The Impact of Perceived Employee–AI Collaboration on Coworker Helping Behaviour

Helping behaviour refers to extra-role behaviour in which employees care for others and assist them with work-related matters within the workplace (Van Dyne & LePine, 1998). Since helping behaviour is not a formal job requirement, whether a coworker provides assistance depends on their overall judgement of the recipient, including whether the employee is worthy of support and trust. According to attribution theory, employee–AI collaboration is not only a technology-use behaviour but also serves as a social cue for coworkers to infer an employee’s effort, willingness to reciprocate, and assumption of responsibility (Reif et al., 2025). Employee–AI collaboration alters, to some extent, the visibility and interpretability of the target employee’s work process to their coworkers (C. Xu et al., 2026). When employees use AI to complete tasks, coworkers see not only the task outcomes but also realise that these outcomes were not generated entirely independently by the employee. Therefore, coworkers must assess whether an employee’s performance reflects their personal effort and ability or AI-enabled support. Because it is difficult for coworkers to fully observe an employee’s latent input during the AI collaboration process, they may infer it through counterfactual comparison: if there were no AI, would the employee still invest the same effort? Would they still assume the same responsibility? This counterfactual comparison reduces employees’ “help-worthiness” in the eyes of their coworkers (L. Xu et al., 2026). If coworkers believe that an employee’s task outcomes are highly dependent on AI while their personal effort, independent ability, and conscientiousness are unclear, continuing to provide help may be seen as condoning low effort or free-riding behaviour (Zhou et al., 2025). Furthermore, because helping co-workers is not an institutional obligation but a voluntary act requiring the investment of time, attention, information, and emotional resources (H. Jia et al., 2021), coworkers may be more inclined to adopt a cautious interaction strategy and reduce proactive assistance when they cannot clearly judge the target employee’s actual mode of engagement in AI collaboration, subsequent interaction needs, and potential responsibility consequences. Therefore, this study proposes the following hypothesis:

**H1.**  *Perceived employee–AI collaboration is negatively related to coworker helping behaviour*.

### 3.2. The Mediating Role of Laziness Attribution

Laziness attribution refers to a dispositional judgement by coworkers that employee–AI collaboration is not intended to improve efficiency but rather to reduce workload, minimise effort, or take shortcuts (Zhou et al., 2025). Attribution theory suggests that when information is incomplete, observers tend to infer an actor’s intrinsic motivation based on visible cues; when behavioural outcomes are attributionally ambiguous, observers are more likely to rely on salient and accessible cues to form internal attributions, thereby judging the actor’s competence and motivation. Although collaborating with AI may be seen as a positive signal of enhanced efficiency and quality in some contexts, when it is difficult for coworkers to observe an employee’s actual effort, AI collaboration is more likely to be interpreted as “taking a shortcut”, triggering laziness attribution and undermining the judgement that the employee is “worthy of help” (Choudhary et al., 2025). Employee–AI collaboration may trigger laziness attribution, not because AI use itself necessarily implies slacking off, but because AI collaboration alters the evidentiary structure through which coworkers judge an employee’s effort. In traditional work settings, coworkers can assess effort levels through the time invested and the problem-solving process; however, in employee–AI collaboration, part of the cognitive labour is shifted to the interaction between the human and the AI, where the employee’s prompt design, result filtering, and content verification are often not fully visible (Raisch & Krakowski, 2021). Conversely, the effects of AI in rapidly generating results, compressing task time, and reducing explicit labour are highly prominent. Consequently, what coworkers see is not the complete employee–AI collaboration process, but a stark contrast between “less visible effort” and “faster task output” (Castelo et al., 2019; Reif et al., 2025). This contrast prompts coworkers to make counterfactual inferences: if the employee did not have AI assistance, would they still have invested enough effort to complete the task? Could they have achieved similar results? If the answer is uncertain, coworkers may discount the employee’s individual effort, attributing successful task outcomes more to AI assistance than to the employee’s own exertion. Especially when AI can handle large amounts of information searching, text writing, data analysis, and proposal generation that originally required the employee’s personal involvement, coworkers are more likely to interpret AI collaboration as a behaviour aimed at reducing personal input, evading hard work, or relying on technology to complete the tasks. Reif et al. (2025) also indicated that employees who receive AI assistance are perceived by peers and even managers as lazier and lacking work passion and initiative compared to those who receive human assistance or no assistance at all. Therefore, this study proposes the following hypothesis:

**H2.**  *Perceived employee–AI collaboration is positively related to coworkers’ laziness attribution toward the focal employee*.

Laziness attribution further undermines coworker helping behaviour. When coworkers attribute employee–AI collaboration to laziness or taking shortcuts, they lower their evaluation of the employee’s effort and work attitude, further weakening the judgement that the employee is “worthy of help.” First, helping behaviour in the workplace is not purely altruistic but a social investment involving resource commitment and uncertainty regarding future returns: helpers must invest time, emotional, and cognitive resources, weighing these against reciprocity expectations and opportunistic risks. They tend to help those who are more likely to reciprocate and less likely to “free-ride” (Zhou et al., 2025). Research on helping behaviour indicates that coworkers are more inclined to assist those perceived as diligent, reliable, and worthy of relational resource investment (Halbesleben & Wheeler, 2015). When coworkers attribute employee–AI collaboration to “laziness/taking shortcuts,” they infer that the employee’s work engagement and contribution are insufficient and that the employee may adopt opportunistic strategies in cooperation. This reduces the judgement of being “worthy of help” and expectations of reciprocity, while increasing the perceived risk of “investment being exploited,” ultimately reducing voluntary coworker helping behaviour. Second, laziness attribution undermines trust in employees. Effort and diligence are key indicators used to evaluate others’ work ethics in professional environments; thus, individuals perceived as having a strong work ethic and diligence are more likely to receive help from others (Woehr et al., 2024). Coworkers are generally more willing to trust and help employees perceived as “norm-abiding and dependable,” while maintaining a cautious and distant attitude toward those perceived as lazy (Choi et al., 2025). When an employee’s collaboration with AI leads to questioning by coworkers, the trust relationship between them is damaged, thereby reducing their willingness to help. Therefore, laziness attribution addresses the question of “whether the employee is worthy of help,” and the inhibitory effect of employee–AI collaboration on coworker helping behaviour may be realised by increasing coworkers’ laziness attribution toward the employee. Therefore, this study proposes the following hypothesis:

**H3.**  *Perceived employee–AI collaboration indirectly reduces coworker helping behaviour through increased laziness attribution*.

### 3.3. The Mediating Role of Responsibility-Avoidance Attribution

Responsibility-avoidance attribution refers to a coworker’s interpretation of an employee’s behaviour as a strategic action aimed at reducing personal responsibility, evading accountability, or creating space for subsequent responsibility shifting (Hargie et al., 2010). Unlike laziness attribution, which focuses on whether an employee invests sufficient effort, responsibility-avoidance attribution focuses on whether an employee is willing to assume clear responsibility for task outcomes (Hu et al., 2026). The key reason why employee–AI collaboration may induce responsibility-avoidance attribution is that AI’s powerful capabilities not only change the way tasks are completed but also the responsibility structure of task outcomes (Bockstedt & Buckman, 2026). In traditional tool-use contexts, tools are typically viewed as an extension of the employee’s actions, and the employee is still considered the primary subject responsible for task judgements and outcomes. However, in AI collaboration contexts, AI can automate tasks, participate in complex decision-making, and influence final solutions, causing task outcomes to exhibit distinct characteristics of human–AI co-production (Doshi & Hauser, 2024). Furthermore, shifting responsibility to AI does not require adherence to the norms of interpersonal interactions, making AI a highly attractive target for responsibility evasion. In such cases, it becomes difficult for coworkers to determine whether key outcomes originated from the employee or from AI output. The more deeply AI is involved in a task, the more interpretive leeway employees have. When the results are positive, employees can showcase the final achievement; when the results are poor or involve complex decision-making risks, employees may gain substantive benefits by shifting responsibility through AI (L. Xu et al., 2026). L. Xu et al. (2026) also indicated that individuals are more inclined to delegate decisions involving high trade-off difficulty to AI. Consequently, coworkers may suspect that employees are using AI to blur responsibility boundaries and reduce personal accountability. Responsibility-avoidance attribution also has a counterfactual basis. Coworkers might imagine: if there were no AI intervention, would the employee have to bear clearer personal responsibility for task judgements and outcomes? It is within this comparison that employee–AI collaboration may be interpreted by coworkers as a strategic behaviour to reduce responsibility. Especially when AI is involved in tasks characterised by high uncertainty or requiring professional judgement, coworkers are more likely to believe that the employee is not merely using AI to improve efficiency but is utilising AI to shirk responsibility and lower personal accountability.

**H4.**  *Perceived employee–AI collaboration is positively related to coworkers’ responsibility-avoidance attribution toward the focal employee*.

This study further argues that responsibility-avoidance attribution inhibits helping behaviour. Responsibility-avoidance attribution emphasises that the actor has a strategic motivation to reduce accountability, which significantly alters the helper’s judgement regarding “whether helping is safe.” On the one hand, helping behaviour is characterised by voluntariness and reciprocity; coworkers are generally more willing to help those who are conscientious (Hargie et al., 2010). Conversely, responsibility-avoidance attribution is interpreted by coworkers as an opportunistic tendency, seeking to gain the output advantages brought by AI while attempting to weaken personal accountability when outcomes are poor (L. Xu et al., 2026). This inference erodes trust and expectations of reciprocity, leading coworkers to believe that “investing in help may not be rewarded and might even be exploited,” thereby reducing resource-based, informational, and emotional assistance. Consistent with this, research by Zhou et al. (2025) indicates that coworkers make moral evaluations of an employee’s AI use and adjust their helping responses accordingly. On the other hand, responsibility-avoidance attribution reinforces coworkers’ expectations of joint liability risks. In employee–AI collaborative hybrid teams, helping often involves sharing information and co-assuming responsibility for processes and outcomes. Once coworkers perceive that an employee tends to attribute failures to AI, they worry about becoming potential “joint responsibility bearers” or “scapegoats” after providing help, leading them to withdraw assistance out of self-protection. Weiner et al. (1988) also pointed out that when others’ predicaments or unfavourable outcomes are seen as “controllable and accountable,” observers are more likely to experience negative emotions such as anger and withdraw helping behaviour. Therefore, responsibility-avoidance attribution addresses the question of “helping riskiness,” further inhibiting coworkers’ helping behaviour by increasing the helper’s risk perception and self-protection motivation. This study proposes the following hypothesis:

**H5.**  *Coworker perceived employee–AI collaboration indirectly reduced coworker helping behaviour through increased responsibility-avoidance attribution*.

### 3.4. The Moderating Role of Human–AI Task Interdependence

Human–AI task interdependence refers to the extent to which employee tasks and AI tasks are mutually dependent and embedded within the workflow, input–output processes, and formation of outcomes (Van der Vegt & Janssen, 2003). Unlike AI usage frequency, AI usage intensity, or employees’ subjective dependence on AI, human–AI task interdependence emphasises the coupling relationship at the level of task structure (Tan & Li, 2025). This study argues that human–AI task interdependence, as a task-contextual contingency factor, influences how coworkers interpret the behavioural cue of employee–AI collaboration. In all-human collaboration contexts, task interdependence enhances the sense of responsibility and willingness to cooperate among members. However, in employee–AI collaboration contexts, AI has evolved from a passive tool into an agentic entity capable of making autonomous decisions under uncertainty, even exerting a reverse influence on employees’ subsequent judgements and actions (Stelmaszak et al., 2025; Tan & Li, 2025). Meanwhile, AI is not a subject of responsibility in the traditional sense; when task outcomes are jointly generated by employees and AI, the boundaries between employee input, AI contribution, and final responsibility become more difficult for coworkers to clearly identify (Castelo et al., 2019). Therefore, the higher the human–AI task interdependence, the more likely the behavioural cue of employee–AI collaboration is to exhibit stronger attributional ambiguity.

It should be noted that although coworkers tend to view AI collaboration as a job requirement or organisational norm when tasks are highly dependent on AI, AI governance in real-world organisations often lags behind AI usage (Birkstedt et al., 2023). Many organisations still lack clear institutional arrangements regarding AI collaboration policies, process disclosure, and responsibility allocation (Grote et al., 2026; Zhao et al., 2026). In such cases, the boundaries between employee input, AI contribution, and ultimate responsibility are more likely to blur (Grote et al., 2026). Related research also indicates that AI collaboration may alter the structure of responsibility attribution, making AI a target for responsibility shifting or offloading (L. Xu et al., 2026). Therefore, in contexts where AI collaboration is not yet fully institutionalised, processes are opaque, and responsibility boundaries are unclear, high human–AI task interdependence may amplify coworkers’ doubts about an employee’s effort and accountability, thereby strengthening laziness and responsibility-avoidance attributions.

Specifically, regarding laziness attribution, high human–AI task interdependence reinforces the invisibility of effort in employee–AI collaboration. Laziness attribution primarily involves coworkers’ judgements of an employee’s effort investment and work motivation (Fügener et al., 2026). When task interdependence between the employee and AI is low, the AI typically only participates in auxiliary stages, such as retrieving information, generating preliminary materials, or providing partial suggestions. The employee still needs to independently complete core judgements and final integration, making it easier for coworkers to identify the employee’s individual contribution. In such cases, employee–AI collaboration is not necessarily interpreted as a reduction in effort (Chugh et al., 2014). Conversely, when employees and AI are highly interdependent in the task workflow, AI may be deeply involved in critical stages, such as information analysis, solution generation, and result formation, making employees’ efforts in prompt design, screening, judgement, review, and revision more difficult for coworkers to observe fully (Shrestha et al., 2019). In workplace environments where competition for resources, evaluations, and promotions exists among coworkers, they may be more inclined to cautiously assess whether the target employee has invested sufficient effort (Flavián et al., 2021). Thus, in contexts of high human–AI task interdependence, employee–AI collaboration is more likely to be interpreted by coworkers as reducing effort, relying on AI, or taking shortcuts, thereby strengthening laziness attribution. Therefore, we propose the following hypothesis:

**H6.**  *Human–AI task interdependence positively moderates the relationship between perceived employee–AI collaboration and laziness attribution*.

Regarding responsibility-avoidance attribution, high human–AI task interdependence strengthens the ambiguity of responsibility boundaries in employee–AI collaboration. Responsibility-avoidance attribution primarily involves coworkers’ judgements of an employee’s willingness to assume responsibility and accountability (Malle, 2006). When human–AI task interdependence is low, the relationship between AI output and an employee’s subsequent decisions is typically loose, making the boundaries of individual judgement and final responsibility relatively clear, even if employees use AI, coworkers can easily interpret it as instrumental support. Conversely, when human–AI task interdependence is high, employee inputs, AI outputs, and final task outcomes are nested within each other, meaning that task results no longer stem entirely from individual judgement or automated AI output. In such cases, it becomes more difficult for coworkers to determine whether responsibility for a deviation in results should be attributed to insufficient employee judgement, erroneous AI output, or inadequate cross-checking during the employee–AI collaboration process (Birkstedt et al., 2023; L. Xu et al., 2026). Since coworker helping behaviour may involve responsibility entanglement and risk diffusion, when employee–AI collaboration occurs within highly interdependent tasks, coworkers may be more concerned that the employee is using AI to blur responsibility boundaries or shift blame to the AI system when outcomes are poor (Hohenstein & Jung, 2020). Therefore, in contexts where AI usage norms and responsibility allocation remain unclear, high human–AI task interdependence strengthens the relationship between perceived employee–AI collaboration and responsibility-avoidance attribution. Therefore, we propose the following hypothesis:

**H7.**  *Human–AI task interdependence positively moderates the relationship between perceived employee–AI collaboration and responsibility-avoidance attribution*.

**Figure 1:**
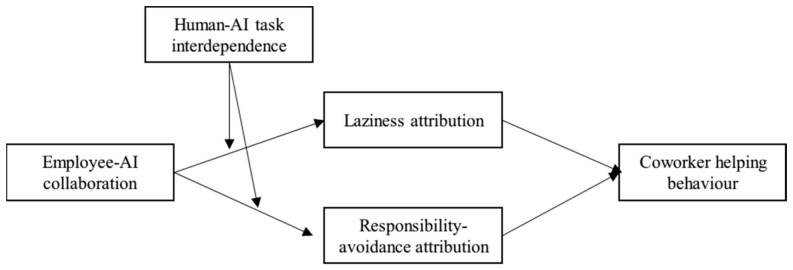
The theoretical model.

The theoretical framework of this study is shown in Figure 1.

### 3.5. Research Overview

To validate the theoretical model, we conducted a questionnaire survey (Study 1) and an experimental study (Study 2). Study 1 employed a two-stage lagged design to reduce reverse causality inference between variables, providing correlational evidence for the theoretical model in a field context. To further clarify the causal relationships between variables, this study utilised a scenario-based experimental design (Study 2) to manipulate employee–AI collaboration and human–AI task interdependence, thereby enhancing internal validity and testing the overall model. The combination of the questionnaire survey and experimental study provides both internal and external validity testing of the research model.

## 4. Study 1: Questionnaire Survey

### 4.1. Sample and Data Collection

This study employed a two-stage time-lagged design to collect questionnaire data. Participant screening was conducted at both the organisational and individual levels. At the organisational level, the study selected enterprises in regions such as Hubei and Jiangsu that had extensively adopted AI applications across their workflows, spanning industries such as finance, IT services, and education. These enterprises applied generative AI, service robots, and algorithmic systems to processes such as decision support, customer service, content production, and process optimisation. At the individual level, participants were required to meet the following criteria: first, they must have formal work experience; second, they must interact with or use AI systems in their daily work; and third, they must be able to identify a coworker with whom they had a collaborative or mutually supportive relationship at work, and whose AI collaboration behaviour was observable, to serve as the subject of evaluation.

To ensure that the two waves of data referred to the same evaluation target, respondents answered questions about the same target coworker at both Time 1 (T1) and Time 2 (T2), with matching conducted using personal IDs. At T1, respondents reported the target coworker’s level of perceived employee–AI collaboration and human–AI task interdependence, along with relevant control variables. At T2, respondents reported their laziness and responsibility-avoidance attribution towards the same target coworker, as well as the extent to which they provided help to that coworker. A total of 450 questionnaires were distributed to the participants. After excluding those with substantial missing data or unsuccessful matching, the final sample comprised 375 valid matched coworker evaluation questionnaires, yielding an effective response rate of approximately 83.3%. The demographic characteristics of the sample are shown in Table 2.

### 4.2. Measurement

The measures used in this study were adapted from established scales in the existing literature to fit the context of employee–AI collaboration in the workplace. This study adopts a coworker observer perspective, necessitating adjustments to some item subjects from ‘focal employee’ to ‘this coworker/the evaluated coworker’ for consistency with the research design. The scales strictly followed the translation and back-translation procedures described earlier. All the items were scored using a 5-point Likert scale.

Employee–AI collaboration: We employed a five-item scale from Kong et al. (2023) adapted from the coworker evaluation perspective. A sample item is: ‘In the workplace, the coworker often uses AI to make task decisions’. Following Zhou et al. (2025), this study measured coworkers’ perceptions of employee–AI collaboration based on observable cues, rather than AI usage logs or actual collaboration frequency. Because this study adopts a coworker-observer perspective, coworker-rated measurement is consistent with the observer-attribution framework. Cronbach’s alpha was 0.943.

Laziness attribution and responsibility-avoidance attribution: Laziness attribution was measured using a three-item scale developed by Zhou et al. (2025). A sample item is: ‘The reason this employee uses AI is that they want to be lazy’. Cronbach’s alpha was 0.891. Responsibility-avoidance attribution was adapted from Tu (2024). A representative item was: ‘This employee collaborates with AI to reduce their own responsibility when the results are unsatisfactory’. Cronbach’s alpha was 0.917.

Coworker helping behaviour: Helping behaviour was measured using five items adapted from Podsakoff et al. (2009). From the coworker observer perspective, the target of help was specified as the evaluated coworker to measure the respondent’s helping behaviour towards that employee. A sample item is: ‘I spent extra time helping this coworker solve work-related problems’. Cronbach’s alpha was 0.936.

Human–AI task interdependence: This study used a three-item task interdependence scale developed by Liden et al. (1997), with wording modified for the employee–AI context: A sample item was, ‘The tasks performed by the employee and the AI are dependent on each other’. Cronbach’s alpha was 0.885.

Control variables: Following Hai et al. (2025) and Zhou et al. (2025), this study included gender, age, education level, work experience, duration of collaboration, relationship quality with coworkers, and employees’ AI usage experience as control variables. Relationship quality with coworkers was included to control for potential interpersonal influences.

### 4.3. Results Analysis

#### 4.3.1. Measurement Model Test

Confirmatory factor analysis (CFA) tested the reliability and discriminant validity of the measurement model, as shown in Table 3. The five-factor model was compared with several competing models. The results indicated that the five-factor model fit the data well (*χ*^2^ = 212.263, *df* = 160, *χ*^2^/*df* = 1.327, CFI = 0.992, TLI = 0.990, SRMR = 0.025, RMSEA = 0.030), with all fit indices meeting the recommended thresholds. By contrast, the fit of the four-, three-, two-, and single-factor models decreased substantially. These results indicate that the five constructs measured in this study are empirically distinguishable and that serious common method bias is unlikely. The standardised factor loadings of all items on their respective latent variables were above 0.60 and significant at the 0.001 level. The AVE values for all constructs exceeded 0.50, and the composite reliability (CR) values were all above 0.70, indicating satisfactory convergent validity. The HTMT index was also examined. As shown in Table 4, all HTMT values were below 0.85, further supporting discriminant validity.

#### 4.3.2. Common Method Biases

This study adopted procedural remedies and statistical techniques to reduce the effects of common method bias (CMB). On the one hand, strategies such as time-lagged measurement and anonymous responding were implemented during the questionnaire design phase. On the other hand, the results of the unmeasured latent method factor technique show that the single-factor model fits poorly (SRMR = 0.119), while the five-factor measurement model before control fits well (SRMR = 0.025). After adding the common method factor, the model fit did not show a substantial improvement (SRMR = 0.025). Moreover, the marker variable technique proposed by Lindell and Whitney (2001) was used for further testing. We selected years of work experience (*r* = −0.034), which had no significant effect on any latent variables and the lowest correlation with the dependent variable as the marker variable. After incorporating this marker variable as a control, the direction and significance of the core paths were substantially unchanged. Therefore, common method bias was not serious.

#### 4.3.3. Descriptive Analysis

Table 5 presents the means, standard deviations, and correlation coefficients of the primary variables. Perceived employee–AI collaboration was significantly and negatively correlated with helping behaviour (*r* = −0.530, *p* < 0.01), indicating that a higher degree of employee–AI collaboration was associated with less helping behaviour. This is consistent with Hypothesis 1. Employee–AI collaboration was positively and statistically significantly correlated with laziness attribution (*r* = 0.624, *p* < 0.01) and responsibility-avoidance attribution (*r* = 0.599, *p* < 0.01), implying that coworkers believe that employee–AI collaboration undermines an individual’s conscientiousness. Similarly, laziness attribution (*r* = −0.562, *p* < 0.01) and responsibility-avoidance attribution (*r* = −0.530, *p* < 0.01) were significantly negatively correlated with coworker helping behaviour; that is, the higher the degree of laziness and responsibility-avoidance attributions by coworkers, the more they tended to reduce helping behaviour. The results in Table 5 show that the square root of the AVE for each construct is greater than its correlation coefficient with the other constructs, satisfying the discriminant validity criteria proposed by Fornell and Larcker (1981).

Notably, a correlation exists between laziness and responsibility-avoidance attribution. Although both stem from social evaluative penalties imposed by coworkers regarding employee–AI collaboration behaviour, their psychological orientations differ: laziness attribution primarily reflects coworkers’ judgement of the employee’s effort investment, while responsibility-avoidance attribution primarily reflects their judgement of the employee’s willingness to assume responsibility. Furthermore, the variance inflation factors (VIF) for laziness attribution (2.004) and responsibility-avoidance attribution (2.017) were both below the critical threshold (3.3), indicating that multicollinearity was not a serious issue. Laziness attribution points toward the front-end process of employee task engagement, serving as a motivational attribution for an unexpended effort. Responsibility-avoidance attribution points toward back-end accountability for task outcomes, centring on accountability for bearing responsibility.

#### 4.3.4. Hierarchical Regression Analysis

We employed hierarchical regression analysis to test the hypotheses, and the results are presented in Table 6. All models control for variables such as coworker gender, age, education level, years of work experience, duration of working together, and the relationship with the target coworker. Model 8 shows that perceived employee–AI collaboration is significantly negatively correlated with coworker helping behaviour (*β* = −0.406, *p* < 0.05), supporting H1. This result echoes that of Zhou et al. (2025), suggesting that employees collaborating with AI lead to negative evaluations of their competence by coworkers, thereby reducing helping behaviour toward those employees. Model 2 indicates that employee–AI collaboration has a significant positive predictive effect on laziness attribution (*β* = 0.551, *p* < 0.01), supporting Hypothesis 2. This indicates that coworkers’ high perception of employee–AI collaboration is significantly and positively correlated with laziness attribution. Furthermore, to test the mediating role of laziness attribution in the relationship between employee–AI collaboration and coworker helping behaviour, this study added the attribution variable to Model 9. The results show that the impact of laziness attribution on coworker helping behaviour is significantly negative (*β* = −0.169, *p* < 0.01), and employee–AI collaboration is still significantly and negatively associated with coworker helping behaviour (*β* = −0.203, *p* < 0.01). This indicates that laziness attribution plays a partial mediating role, supporting Hypothesis 3. The results of Model 5 show that after controlling for relevant variables, employee–AI collaboration has a significant positive impact on responsibility-avoidance attribution (*β* = 0.583, *p* < 0.01); in Model 6, this positive relationship remains significant (*β* = 0.319, *p* < 0.01), supporting Hypothesis 4. This indicates that high perceived employee–AI collaboration is significantly correlated with responsibility-avoidance attribution. On this basis, to test the mediating role of responsibility-avoidance attribution, this study included responsibility-avoidance attribution in the regression model in Model 9. The results show that responsibility-avoidance attribution has a significant negative impact on coworker helping behaviour (*β* = −0.189, *p* < 0.01).

**Figure 2:**
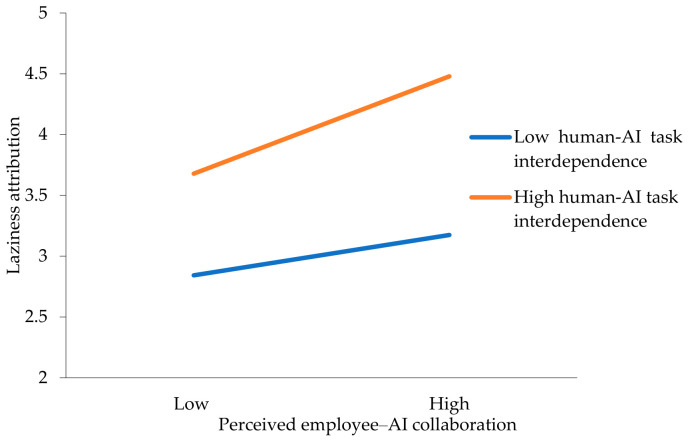
Moderating effect of human–AI task interdependence on the relationship between perceived employee–AI collaboration and laziness attribution.

Hypothesis 6 proposed that human–AI task interdependence positively moderates the relationship between employee–AI collaboration and laziness attribution. To test this moderating effect, this study included an interaction term between employee–AI collaboration and human–AI task interdependence in the regression analysis, with laziness attribution as the dependent variable. The results of Model 3 show that the regression coefficient of the interaction term is positive and statistically significant (*β* = 0.039, *p* < 0.01). This indicates that human–AI task interdependence positively moderates the impact of perceived employee–AI collaboration on laziness attribution. To visually present the moderating effect, a simple slope plot was drawn according to Aiken and West (1991), as shown in Figure 2.

**Figure 3:**
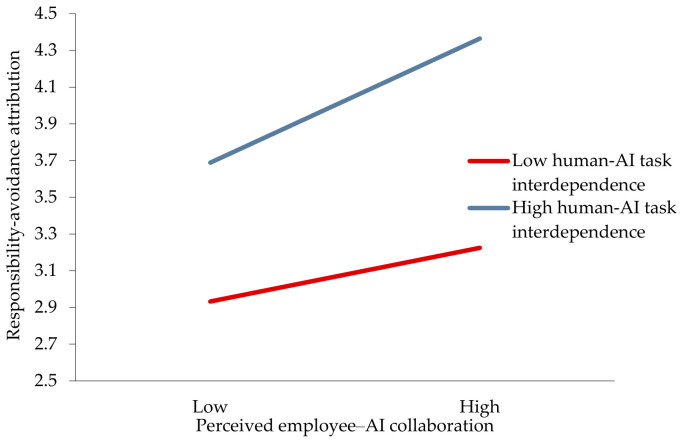
Moderating effect of human–AI task interdependence on the relationship between perceived employee–AI collaboration and responsibility-avoidance attribution.

Similarly, Hypothesis 7 proposed that human–AI task interdependence positively moderates the relationship between employee–AI collaboration and responsibility-avoidance attribution. To test this moderating effect, this study included an interaction term between perceived employee–AI collaboration and human–AI task interdependence in the regression analysis, with responsibility-avoidance attribution as the dependent variable. The results of Model 6 show that the regression coefficient of the interaction term is positive and statistically significant (*β* = 0.065, *p* < 0.01). This indicates that human–AI task interdependence positively moderates the impact of perceived employee–AI collaboration on responsibility-avoidance attribution. A simple slope plot was drawn to visually present the moderating effects (Figure 3).

To test whether human–AI task interdependence moderates the indirect effect of perceived employee–AI collaboration on coworker helping behaviour through laziness attribution, this study conducted a moderated mediation analysis using 5000 bootstrapping samples. The results in Table 7 indicate that when human–AI task interdependence is low (−1 SD), the indirect effect of perceived employee–AI collaboration on coworker helping behaviour via laziness attribution is −0.054, with a 95% confidence interval of [−0.109, 0.003], which includes zero, indicating a non-significant mediation effect. Conversely, when human–AI task interdependence is high (+1 SD), the indirect effect is −0.065, with a 95% confidence interval of [−0.130, −0.015], which does not include zero. Furthermore, the results for responsibility-avoidance attribution show a similar trend. When human–AI task interdependence is low (−1 SD), the indirect effect of employee–AI collaboration on coworker helping behaviour via responsibility-avoidance attribution is −0.076, with a 95% confidence interval of [−0.130, −0.030], excluding zero; when human–AI task interdependence is high (+1 SD), the indirect effect is −0.101, with a 95% confidence interval of [−0.171, −0.039], also excluding zero. These results suggest that when human–AI task interdependence is low, the indirect effect of employee–AI collaboration on laziness attribution is significantly weakened or even non-significant. One possible reason is that laziness attribution is essentially an inference regarding the actor’s effort and work motivation, the formation of which typically requires strong process-related cues. When human–AI task interdependence is low, the task boundaries and interfaces between humans and AI are clearer, and AI is more likely to be perceived as an instrumental auxiliary resource rather than a “collaborative agent” sharing key task components with the employee. Consequently, coworkers tend to evaluate employee performance based on verifiable deliverables and output quality, rather than inferring whether they are “reducing effort” based on cues from an invisible process.

#### 4.3.5. Supplementary Analysis

To further test the robustness of the results, we conducted supplementary analyses using participants’ actual perceptions of the experimental materials from Study 2 as a continuous variable. The results showed that perceived employee–AI collaboration significantly and positively predicted laziness attribution (*β* = 0.426, *p* < 0.01). After further incorporating human–AI task interdependence and the interaction term, this relationship remained significant (*β* = 0.327, *p* < 0.01), and human–AI task interdependence significantly and positively predicted laziness attribution (*β* = 0.314, *p* < 0.01). Meanwhile, perceived employee–AI collaboration also significantly and positively predicted responsibility-avoidance attribution (*β* = 0.362, *p* < 0.01). In the full model, perceived employee–AI collaboration still had a significant positive effect on responsibility-avoidance attribution (*β* = 0.264, *p* < 0.01), and human–AI task interdependence significantly and positively predicted responsibility-avoidance attribution (*β* = 0.303, *p* < 0.01).

## 5. Study 2: Scenario Experiment

### 5.1. Participants

To further examine the causal relationships among the variables, this study recruited full-time employees through the Credamo platform to participate in an online experiment. The goal was to test the causal impact of employee–AI collaboration on helping behaviour and its underlying psychological mechanisms. Study 2 primarily serves to verify the causal effect of employee–AI collaboration on the willingness to help the target coworker, providing supplementary evidence for the findings of Study 1. The following screening criteria were established to select eligible participants: (1) at least one year of full-time work experience; (2) the participant’s organisation has adopted AI technology; and (3) the participant’s daily work requires collaboration with coworkers or involves scenarios of mutual assistance. To ensure data quality, an attention check item was included in the experimental questionnaire, such as “This is an attention check item; please select ‘Strongly Disagree’ for this question.” A final valid sample of 320 participants was obtained.

### 5.2. Experimental Design and Procedure

This study employed a 2 (Employee–AI collaboration: high vs. low) × 2 (Human–AI task interdependence: high vs. low) between-subjects factorial design. A scenario-based experiment was conducted, with participants randomly assigned to one of four experimental conditions. At the start of the experiment, participants provided demographic information. To ensure that participants could distinguish between AI technology in the workplace and traditional technologies (e.g., computer or automation technology), we followed Tang et al. (2023) by presenting them with a formal definition of AI technology applications. Subsequently, variables were manipulated through a situational narrative involving a hypothetical employee named Zhang San. Finally, participants were asked to adopt the role of a coworker (observer) to evaluate their attributions of laziness and responsibility-avoidance toward this employee (Zhang San) and to report their own helping behaviour. Upon completion of the experiment, participants were informed that the study was conducted solely for research purposes.

The manipulation of the degree of employee–AI collaboration was adapted from the definitions and operational materials regarding AI technology application by Tang et al. (2023) and Zhou et al. (2025). At the beginning of the experiment, participants were asked to assume the role of a “coworker/observer” and read a short description of a virtual employee (Zhang San) to manipulate the perceived level of employee–AI collaboration. The scenario was set in a typical team task (such as client proposals, service responses, or data analysis reports) to ensure that “coworkers could observe cues of the other party’s collaboration with AI.” For instance, the interaction process with AI and the method of integrating outputs would be visible within a project collaboration platform or shared document.

In the high employee–AI collaboration condition, participants read a description of Zhang San frequently interacting with AI: “You notice that your coworker Zhang San frequently collaborates with AI during work to complete various tasks, including writing reports and processing data. He engages in multiple rounds of interaction, revision, and integration with the AI throughout the task process.”

In the low employee–AI collaboration condition, participants read a description of Zhang San rarely interacting with AI: “You notice that your coworker, Zhang San, rarely collaborates with AI during work; AI only appears in a few auxiliary tasks, such as information retrieval.”

The manipulation of human–AI task interdependence was adapted from the experimental materials developed by Tan and Li (2025). To avoid confusion with employee–AI collaboration, this study defines human–AI task interdependence as a characteristic at the task structure level.

High human–AI task interdependence: The various stages of this task are closely linked, and there is a strong task interdependence between Zhang San and the AI. The AI’s analytical results influence Zhang San’s subsequent judgements and solution choices, while Zhang San’s supplementary information regarding data, goals, and constraints also affects the AI’s subsequent outputs.

Low human–AI task interdependence: The various stages of this task are relatively independent. The AI’s output does not directly determine Zhang San’s subsequent work, nor does his progress depend on it. Since the task components are decoupled, the final work product is decided upon and completed by Zhang San.

### 5.3. Measurements

The measurement variables in this study used established scales from the literature, appropriately adapted to fit the employee–AI collaboration context and the coworker evaluation perspective. First, the general work behaviour descriptions in the original scales were adjusted to reflect employee–AI collaboration scenarios. Second, the self-evaluation perspective was shifted to a coworker evaluation perspective. Third, while preserving the original meaning of the constructs, the wording was simplified to improve clarity for participants. Prior to the formal study, a pretest was conducted, and certain expressions were refined based on participant feedback. Organisational behaviour experts were invited to evaluate the content validity of the revised items. The pretest results indicated that the items were clearly phrased and the scale reliability reached acceptable levels, suggesting that the revised scales were suitable for the context of this study. The scenario experiment used the same scales as the questionnaire survey, with all scales scored using a 5-point Likert scale. Regarding coworker helping behaviour, as the scenario experiment could not directly observe actual helping behaviour, following the research of Zhou et al. (2025), helping intention was used as a proxy indicator for helping behaviour to measure the extent to which participants were willing to provide assistance to the target employee in the given scenario. Descriptive statistics and correlation coefficients (experience) is presented in Table 8.

### 5.4. Manipulation Check

At the end of the experiment, we set up two manipulation checks to verify whether the experimental materials effectively manipulated employee–AI collaboration and human–AI task interdependence. First, participants used the employee–AI collaboration scale from Study 1 to evaluate the degree of AI collaboration of the situational character “Zhang San.” Cronbach’s α for this scale was 0.910. Second, the participants used the human–AI task interdependence scale from Study 1 to evaluate the degree of interdependence between Zhang San and the AI during task completion. Cronbach’s α for this scale was 0.907.

### 5.5. Research Results

#### 5.5.1. Manipulation Test Results

The results of the independent samples t-test indicated that participants in the high employee–AI collaboration group perceived the target employee’s collaboration with AI to be stronger (M = 3.86, SD = 0.73), which was significantly higher than that in the low employee–AI collaboration group (M = 2.30, SD = 0.80). There was a significant difference between the two groups, *t*(318) = 18.32, *p* < 0.001, Cohen’s d = 2.05. Meanwhile, the high human–AI task interdependence group (M = 3.92, SD = 0.85) scored significantly higher than the low human–AI task interdependence group (M = 1.83, SD = 0.76), with a significant difference between the two groups, *t*(318) = 23.17, *p* < 0.001, Cohen’s d = 2.59. The manipulation check results demonstrated that the experimental materials effectively distinguished between high and low employee–AI collaboration and task interdependence scenarios.

#### 5.5.2. Hypothesis Test Results

Main effect test: This study employed ordinary least squares (OLS) regression to analyse the 2 × 2 between-subjects experimental data. When testing willingness to help, variables such as gender, age, education level, industry, years of work experience, and duration of working together were controlled. The regression results, as shown in Table 9, indicate that employee–AI collaboration has a significant negative impact on coworker helping behaviour (*b* = −0.504, *t* = −3.187), supporting Hypothesis H1. After further incorporating laziness and responsibility-avoidance attributions, the direct effect of employee–AI collaboration on willingness to help the coworker remained significantly negative (*b* = −0.323, *t* = −2.026). This suggests that employee–AI collaboration serves as a behavioural cue that triggers attributional judgements, and laziness attribution is the judgement formed by participants based on this cue, rather than a result directly manipulated in the experimental materials. Furthermore, employee–AI collaboration had a significant positive impact on laziness attribution (*b* = 1.005, *t* = 5.550), supporting Hypothesis H2; compared to low AI collaboration scenarios, the laziness attribution toward the target employee was higher in employee–AI collaboration scenarios. Employee–AI collaboration also had a significant positive impact on responsibility-avoidance attribution (*b* = 0.445, *t* = 2.565), supporting H4.

**Figure 4:**
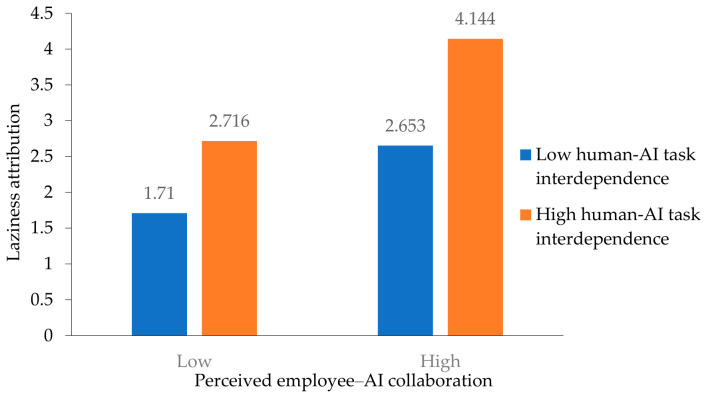
Moderating effect of human–AI task interdependence on perceived employee–AI collaboration and laziness attribution (experiment).

**Figure 5:**
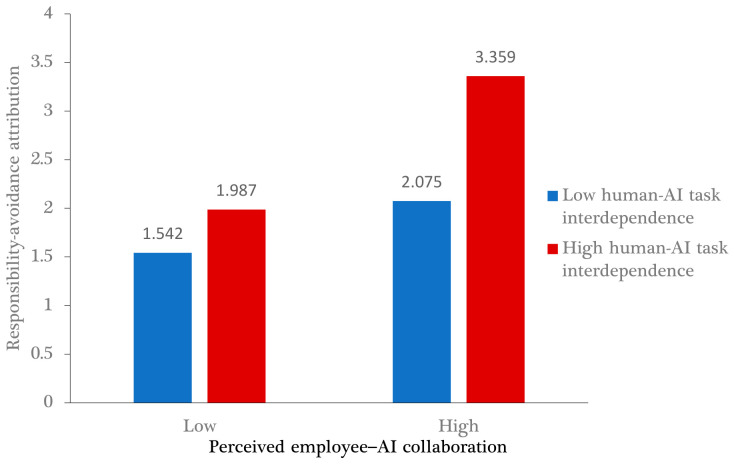
Moderating effect of human–AI task interdependence on perceived employee–AI collaboration and responsibility-avoidance attribution (experiment).

Moderated effect test. For laziness attribution, the interaction term of AI × TI was positive and statistically significant (*b* = 0.486, *t* = 2.894), indicating that in contexts of high human–AI task interdependence, employee–AI collaboration is more likely to trigger laziness attribution from coworkers toward that employee. Further simple slope analysis results show that under conditions of low human–AI task interdependence, the impact of employee–AI collaboration on laziness attribution is significant (*b* = 1.01, *t* = 5.55), whereas under conditions of high human–AI task interdependence, this effect is strengthened (*b* = 1.491, *t* = 8.227). For responsibility-avoidance attribution, human–AI task interdependence positively moderated the relationship between employee–AI collaboration and responsibility-avoidance attribution (*b* = 0.839, *t* = 3.417), supporting Hypothesis 7. Simple slope analysis indicates that under low human–AI task interdependence, the impact of employee–AI collaboration on responsibility-avoidance attribution is *b* = 0.445 (*t* = 2.565); under high human–AI task interdependence, this impact increases to *b* = 1.283 (*t* = 7.400). Figure 4 and Figure 5 visually present the aforementioned interaction relationships.

Mediating effect test: We used the bootstrapping procedure to test the parallel mediating roles of laziness and responsibility-avoidance attributions. As shown in Table 10, the indirect effect of employee–AI collaboration on coworker helping behaviour through laziness attribution was −0.187, 95% CI = [−0.365, −0.044]; the indirect effect through responsibility-avoidance attribution was −0.225, 95% CI = [−0.370, −0.095]. The total indirect effect was −0.412, 95% CI = [−0.598, −0.256], and the total effect was −0.984, 95% CI = [−1.225, −0.742]. This indicates that both indirect paths were statistically supported. Decomposing the indirect effects shows that responsibility-avoidance attribution (0.225) is greater than that of the laziness attribution (0.187), suggesting that in the context of employee–AI collaboration, coworkers are more concerned that employee–AI collaboration blurs responsibility boundaries, thereby reducing helping behaviour. A possible reason for this is the relational risk attributes of coworker helping behaviour. Laziness attribution primarily affects coworkers’ evaluations of the target employee’s effort level and work attitude, whereas responsibility-avoidance attribution further touches upon the potential risks to the helpers themselves (Hu et al., 2026). Considering that coworker helping behaviour is voluntary, resource-consuming, and risk-sensitive (Halbesleben & Wheeler, 2015), the motivation for coworkers to reduce help is stronger when employee–AI collaboration is interpreted as blurring responsibility boundaries. In highly competitive workplace contexts, employees tend to evaluate the relational benefits and potential risks of helping behaviour more cautiously, and a competitive climate also weakens the promoting effect of prosocial identity on interpersonal helping (David et al., 2021).

Moderated mediating effect test: This study utilised the bootstrap method to estimate conditional indirect effects under different levels of human–AI task interdependence, with the results presented in Table 11. Under the condition of low human–AI task interdependence, the indirect effect of employee–AI collaboration on helping behaviour through increased laziness attribution was not significant (β = −0.092, Boot SE = 0.065, 95% CI = [−0.224, 0.029], including 0). However, the indirect effect of increased responsibility-avoidance attribution was significantly negative (β = −0.129, Boot SE = 0.040, 95% CI = [−0.177, −0.021]). Under the condition of high human–AI task interdependence, both indirect effects were significant and stronger: the indirect effect for the laziness attribution path was (β = −0.136, Boot SE = 0.098, 95% CI = [−0.345, −0.041]), and the indirect effect for the responsibility-avoidance attribution path was (β = −0.258, Boot SE = 0.095, 95% CI = [−0.452, −0.080]). Therefore, H5 was supported, and H3 received conditional support.

## 6. Conclusion and Discussion

### 6.1. Main Findings

With the widespread adoption of robots, algorithms, and artificial intelligence (AI) in organisational settings, collaboration between AI technologies and human employees has become increasingly prevalent. Drawing on attribution theory and an interpersonal relations perspective, this study examined the mechanisms and boundary conditions through which perceived employee–AI collaboration affects coworker helping behaviour. First, perceived employee–AI collaboration is negatively related to coworker helping behaviour. Second, laziness and responsibility-avoidance attributions mediated the relationship between employee–AI collaboration and coworker helping behaviour, with the effect of responsibility-avoidance attribution being stronger than that of laziness attribution. Third, human–AI task interdependence positively moderated the relationship between perceived employee–AI collaboration and attributional mechanisms. When human–AI task interdependence is high, coworkers are more likely to interpret employee–AI collaboration as laziness or responsibility-avoidance, thereby strengthening its negative spillover effects.

### 6.2. Theoretical Contributions

First, this study extends the research on social punishment for AI use from general evaluation scenarios to coworker interaction scenarios within organisations and expands attribution theory from traditional interpersonal interactions to human–machine hybrid task scenarios. Traditional attribution theory primarily focuses on how observers infer an actor’s effort, intention, and responsibility based on human behavioural cues (Carson et al., 2024; Malle, 2006). However, in the context of employee–AI collaboration, observers no longer face simple human behaviour but rather hybrid behavioural cues composed of the employee, AI, and final outcome (Hu et al., 2026; Reif et al., 2025). This study demonstrates that coworkers form negative attributions around employee–AI collaboration, thereby triggering AI-dependence stigmatisation judgements, which expands attribution theory from traditional interpersonal interactions to human–AI hybrid task scenarios. Furthermore, while existing research on AI social evaluation penalties mostly focuses on general contexts (Claessens et al., 2025; Reif et al., 2025), this study finds that employee–AI collaboration inhibits coworker helping behaviour, indicating that AI penalties not only trigger low social evaluations but also further reduce helping behaviour (Earp et al., 2024). By linking the social evaluation penalty of AI collaboration to coworker helping behaviour in competitive workplace contexts, this study enriches the study of impression management and workplace helping behaviour in the context of AI collaboration.

Second, this study examines the mediating mechanisms of laziness and responsibility-avoidance attribution, further revealing two heterogeneous attribution logics at the front end of perceived morality. Although Zhou et al. (2025) explained why coworkers reduce help from the perspective of overall perceived morality, it is difficult to clarify exactly how moral perceptions arise or to directly translate these findings into managerial interventions. This study traces the antecedent formation mechanisms of perceived morality: laziness attribution and responsibility-avoidance attribution. This indicates that the moral perception of AI users does not emerge out of thin air but originates from two more specific attributional judgements: helping reciprocity and helping risk, thereby providing more actionable leverage for AI governance. More importantly, existing research mostly explains AI usage penalties through dimensions such as ability/effort (Reif et al., 2025) and authenticity (Sahebi et al., 2026). This study further finds that the absolute value of the indirect effect of the responsibility-avoidance attribution path is greater than that of the laziness attribution path. In workplace contexts, coworkers focus not only on the target employee’s ability or effort but, more importantly, on the potential liability risks brought about by their assistance. Consequently, this study reveals a relatively overlooked responsibility transfer mechanism in employee–AI collaboration contexts, thereby advancing the explanation of AI social penalties from effort/ability evaluations to the level of responsibility attribution.

Third, this study extends task interdependence from traditional all-human collaboration contexts to employee–AI collaboration contexts, revealing the boundary conditions for the negative interpersonal consequences of employee–AI collaboration. In all-human collaboration contexts, higher task interdependence can enhance the sense of responsibility and agency among members, thereby promoting interpersonal interaction and willingness to cooperate to complete the work (Grabner et al., 2022). However, in employee–AI collaboration, task interdependence can exacerbate attributional ambiguity. When human and machine tasks are highly coupled, and the AI collaboration process lacks transparency or clear boundaries of responsibility, the lines between employee input, AI output, and final results become more difficult to distinguish. This increases uncertainty in coworkers’ judgements regarding an employee’s actual effort and accountability, which in turn amplifies negative attributions and weakens coworker helping behaviour. Furthermore, Tan and Li (2025) revealed the moderating effect of human–AI interdependence on AI use and employee moral disengagement from the perspective of the AI user. From an observer’s perspective, this study finds that coworkers’ attributional reactions to employee–AI collaboration exhibit a clear asymmetry: under low task interdependence conditions, AI is more easily understood as an auxiliary tool; under high interdependence conditions, AI collaboration is more likely to be negatively attributed by coworkers. This finding suggests that social evaluation penalties are not an inevitable outcome of AI collaboration but depend on task interdependence structures. Consequently, this study extends task interdependence theory from an observer’s perspective (Castelo et al., 2019; Shrestha et al., 2019), demonstrating that human–AI task interdependence has conditional social evaluation effects in employee–AI collaboration scenarios.

### 6.3. Managerial Implications

First, when promoting the implementation of AI, managers should expand the scope of AI collaborative governance from a technical issue of “improving efficiency” to an interpersonal issue of “maintaining team mutual aid relationships.” This study finds that coworkers’ perception of employee–AI collaboration reduces helping behaviour, indicating that AI collaboration not only affects individual performance but also generates negative interpersonal spillovers. Therefore, organisations should not merely require employees to use AI; they must also establish the legitimacy of AI collaboration through institutionalised explanations and team communication. Specifically, managers can clarify which tasks are suitable for AI and which stages require human judgement and review. Through training and case sharing, they can demonstrate the actual effort employees invest in prompt design, information filtering, result revision, and quality control, ensuring that AI collaboration is understood as an extension of professional competence rather than “taking a shortcut.” Simultaneously, organisations should avoid moralising or stigmatising AI use. Instead, they should encourage employees to openly and moderately disclose how AI is involved, fostering a rational, transparent, and responsible atmosphere for AI collaboration. This approach aims to unlock the efficiency benefits of AI while reducing coworkers’ negative evaluations of AI users and their withdrawal of help.

Second, managers should design interventions targeting laziness attribution and responsibility-avoidance attribution separately, prioritising the clarification of the chain of responsibility. This study indicates that the key to how employee–AI collaboration affects coworker helping behaviour is not merely a general perception that the AI user is “less moral” but rather the formation of two specific judgements: first, that the user has reduced their effort, and second, that they may be shifting or evading responsibility. Moreover, the indirect effect of the responsibility-avoidance attribution path was relatively strong. Therefore, organisations should, on one hand, increase the visibility of effort, for example, by requiring employees to briefly explain the AI-involved stages, manual revisions, and review criteria in important collaborative tasks, allowing coworkers to see the cognitive labour behind AI collaboration. On the other hand, it is even more crucial to clarify the attribution of responsibility for human–machine joint outputs. For instance, it could be stipulated that AI output serves only as a suggestion, while final judgements, external commitments, and risk-taking remain the responsibility of the specific position or person in charge. For tasks such as customer commitments, compliance documents, complaint handling, and high-sensitivity decision-making, methods like process logging, manual review, and responsibility confirmation can be adopted; for low-risk, low-interdependence tasks, sampling-based reviews or brief records can be used to prevent governance measures from becoming a new collaborative burden.

Third, managers should implement differentiated governance through grading interdependence and modular design. High human–AI task interdependence does not inherently lead to negative attribution; the key lies in whether employee–AI collaboration is transformed into organisational norms characterised by transparency and clear accountability. Therefore, organisations should transition AI collaboration from ambiguous individual practices to institutionalised workflows. For high-interdependence tasks, where AI is deeply embedded in team collaboration, client delivery, or cross-departmental processes, organisations should pre-design clear task boundaries and responsibility nodes. This involves specifying which stages are AI-assisted and which must be judged, reviewed, and finalised by employees, thereby reducing attribution ambiguity through a framework of “process documentation + key node review + final responsible person confirmation.” For low-interdependence tasks, flexibility should be maintained by utilising lightweight recordings or post hoc spot checks. When necessary, managers can also use modular design to break down highly interdependent tasks into identifiable, reviewable, and accountable sub-tasks, reducing responsibility confusion between employees, AI, and final outcomes. Through differentiated governance, organisations can transform AI collaboration into team-recognised work norms, thereby mitigating damage to peer-helping relationships.

### 6.4. Limitations and Future Research

First, this study focused on negative attribution mechanisms to explain the decline in helping behaviour but did not deny that employee–AI collaboration may also trigger positive attributions. Future research could adopt a more balanced perspective to explore how perceived effort and perceived agency operate as parallel mediators affecting coworker helping behaviour. Second, this study treats employee–AI collaboration as a relatively holistic concept and does not sufficiently distinguish between different AI types. Future research could further compare the social-evaluative consequences of collaborating with different kinds of AI and examine whether AI autonomy, explainability, transparency, and anthropomorphism affect laziness attributions, responsibility-avoidance attributions, and helping behaviour. Third, this study captures perceived employee–AI collaboration rather than the complete objective process of employee–AI collaboration. While this approach aligns with the observer attribution perspective, it limits inferences regarding actual employee–AI collaboration. Future research could utilise multi-source data, such as employee self-assessments and AI collaboration logs, for analysis. Additionally, although this study employed a two-stage survey to mitigate common method bias, both the mediator and dependent variables in the questionnaire were provided by the same respondents, which may still fail to eliminate the influence of evaluators’ overall attitudes and existing relationship quality. Future research could utilise quasi-natural experimental data to examine the impact of employee–AI collaboration on actual helping behaviour in natural organisational settings. Fourth, the sample in this study mainly comprised Chinese organisations. Whether these conclusions apply to environments with low power distance, high individualism, or more mature AI governance norms remains unclear. Future research could use cross-cultural samples to compare how employee–AI collaboration affects coworkers’ attributions and helping behaviour across different cultural backgrounds.

## Figures and Tables

**Table 1:** Studies related to employee–AI collaboration.

Author (Year)	Theoretical Foundation	Mediating Variables	Dependent Variables	Subjects	Effects
Man Tang et al. (2022)	Complementarity theory and role theory	Role breadth self-efficacy, role ambiguity	Job performance	AI users	Positive effect
Senoner et al. (2024)	Self-determination theory	None	Task performance	AI users	Positive effect
Tang et al. (2023)	Social belonging model	Need for belonging and loneliness	Helping behaviour, drinking, and work depression	AI users	Negative effect
N. Jia et al. (2024)	Employee creativity theory	Conservation of cognitive resources, job complexity, and challenges	Employee creativity	Sales staff	Double-edged effect
Huang and Gursoy (2024)	Transactional theory of stress	Thriving at work, job insecurity	Proactive service behaviour	Service employees	Double-edged effect
Hai et al. (2025)	JD-R model	Work alienation	Employee equity behaviour	AI users	Negative effect
Tan and Li (2025)	Social cognitive theory	Perceived moral disengagement	Counterproductive work behaviour	AI users	Negative effect
Bai et al. (2025)	Event system theory and cognitive-affective processing system theory	Job crafting	Service performance	AI users	Negative effect
Zhou et al. (2025)	Social cognitive theory	Perceived morality	Helping behaviour	Coworker perspective	Negative effect
Reif et al. (2025)	Attribution theory	None	Laziness	Observer perspective	Negative effect
This study	Attribution theory	Laziness and responsibility-avoidance attribution	Helping behaviour	Coworker perspective	Negative spillover effect

**Table 2:** Demographic analysis of the sample.

Variables	Category	Number	Percentage	Variables	Category	Number	Percentage
Gender	Male	182	48.50%	Duration of collaboration	≤0.5 year	14	3.70%
	Female	193	51.50%		0.5–1 year	33	8.80%
Age	Under 20 years	72	19.20%		1–3 years	133	35.50%
	20–30 years	175	46.70%		3–5 years	124	33.10%
	30–40 years	101	26.90%		≥5 years	71	18.90%
	Over 40 years	27	7.20%	Relationship quality with coworkers	Very poor	9	2.40%
Education	Senior high school or below	52	13.90%		Poor	29	7.70%
	College	105	28%		General	98	26.10%
	Undergraduate	161	42.90%		Better	162	43.20%
	Postgraduate	57	15.20%		Very good	77	20.50%
Work experience	≤1 year	19	5.10%	Frequency of AI usage	≤25%	26	6.90%
	1–3 years	71	18.90%		26–50%	130	34.70%
	3–5 years	95	25.30%		51–75%	118	31.50%
	5–10 years	121	32.30%		≥76%	86	22.90%
	≥10 years	69	18.40%		Almost all	15	4.00%

**Table 3:** Confirmatory factor analysis.

Model	χ^2^	df	χ^2^/df	CFI	TLI	SRMR	RMSEA
Five-Factor Model	212.263	160	1.327	0.992	0.990	0.025	0.030
Four-Factor Model	422.333	164	2.575	0.960	0.954	0.039	0.065
Three-Factor Model	1205.811	167	7.220	0.840	0.818	0.072	0.129
Two-Factor Model	1679.165	169	9.936	0.767	0.738	0.102	0.155
One-Factor Model	2538.666	170	14.933	0.634	0.591	0.119	0.193

*Note*. Four-factor model (merging laziness attribution and responsibility-avoidance attribution), three-factor model (further merging employee–AI collaboration and two types of attribution), two-factor model (merging coworker helping behaviour and human–AI task interdependence), and single-factor model (combining all constructs).

**Table 4:** HTMT index test.

Construct Relationship	HTMT	Boot SE	95% LLCI	95% ULCI
Employee–AI collaboration–laziness attribution	0.680	0.034	0.611	0.744
Employee–AI collaboration–responsibility-avoidance attribution	0.645	0.032	0.582	0.706
Employee–AI collaboration–coworker helping behaviour	0.565	0.045	0.474	0.651
Employee–AI collaboration–human–AI task interdependence	0.500	0.046	0.410	0.588
Laziness attribution–responsibility-avoidance attribution	0.809	0.028	0.754	0.863
Laziness attribution–coworker helping behaviour	0.614	0.039	0.535	0.687
Laziness attribution–human–AI task interdependence	0.540	0.053	0.435	0.641
Responsibility-avoidance attribution–coworker helping behaviour	0.574	0.040	0.491	0.650
Responsibility-avoidance attribution–human–AI task interdependence	0.489	0.052	0.384	0.588
Responsibility-avoidance attribution–human–AI task interdependence	0.547	0.045	0.458	0.633

**Table 5:** Descriptive statistics and correlation analysis.

	Mean	St.d	1	2	3	4	5	6	7	8	9	10	11
Gender (1)	1.515	0.500											
Age (2)	2.221	0.838	0.085										
Education (3)	2.595	0.908	0.078	−0.138 **									
Work experience (4)	2.949	1.230	−0.005	0.278 **	0.041								
Duration of collaboration (5)	3.067	1.303	−0.098	0.283 **	−0.140 **	0.114 *							
Relationship quality with coworkers (6)	3.035	1.134	0.063	0.026	0.029	−0.020	0.270 **						
Employee–AI collaboration (7)	3.289	1.096	0.104 *	−0.026	0.128 *	0.001	−0.231 **	−0.247 **	*0.877*				
Laziness attribution (8)	3.193	1.057	0.028	−0.021	0.086	0.098	−0.256 **	−0.304 **	0.624 **	*0.857*			
Responsibility-avoidance attribution (9)	3.250	1.111	−0.007	0.007	0.052	0.009	−0.178 **	−0.258 **	0.599 **	0.631 **	*0.860*		
Coworker helping behaviour (10)	2.632	1.184	−0.069	0.044	−0.097	−0.034	0.360 **	0.576 **	−0.530 **	−0.562 **	−0.530 **	*0.867*	
Human–AI task interdependence (11)	3.024	1.113	0.032	−0.086	0.071	−0.060	−0.288 **	−0.237 **	0.457 **	0.480 **	0.440 **	−0.499 **	*0.850*

*Note*. Italicized diagonal values are the square roots of the AVE, and the off-diagonal values are Pearson’s correlation coefficients. ** *p* < 0.01, and * *p* < 0.05.

**Table 6:** Hierarchical regression results.

Variables	Laziness Attribution			Responsibility-Avoidance Attribution				Coworker Helping Behaviour		
	M 1	M 2	M 3	M 4	M 5	M 6	M 7	M 8	M9	M10
Gender	0.039	−0.071	−0.054	−0.031	−0.148	−0.127	−0.175	−0.094	−0.134	−0.122
	(0.377)	(−0.831)	(−0.658)	(−0.278)	(−1.577)	(−1.382)	(−1.781)	(−1.066)	(−1.595)	(−1.498)
Age	0.022	−0.001	0.003	0.075	0.051	0.054	−0.033	−0.017	−0.007	−0.013
	(0.329)	(−0.015)	(0.049)	(1.035)	(0.851)	(0.920)	(−0.529)	(−0.294)	(−0.132)	(−0.243)
Education	0.073	0.001	0.004	0.061	−0.016	−0.011	−0.103	−0.049	−0.052	−0.045
	(1.276)	(0.012)	(0.078)	(0.979)	(−0.306)	(−0.207)	(−1.891)	(−1.004)	(−1.120)	(−1.002)
Duration of collaboration	0.093	0.092	0.104	0.001	0.000	0.015	−0.036	−0.036	−0.020	−0.024
	(1.132)	(1.096)	(1.002)	(0.026)	(0.012)	(0.386)	(−0.889)	(−0.982)	(−0.576)	(−0.686)
Working relationship	−0.157 **	−0.084 *	−0.058	−0.108 *	−0.031	−0.008	0.191 **	0.137 ***	0.117 ***	0.089 *
	(−3.644)	(−2.363)	(−1.653)	(−2.309)	(−0.787)	(−0.218)	(4.687)	(3.725)	(3.324)	(2.558)
Relationship quality with coworkers	−0.236 **	−0.122 **	−0.103 **	−0.222 **	−0.101 *	−0.083	0.550 **	0.466 ***	0.426 ***	0.415 **
	(−5.000)	(−3.104)	(−2.682)	(−4.325)	(−2.340)	(−1.956)	(12.349)	(11.465)	(10.901)	(10.910)
Employee–AI collaboration		0.551 **	0.357 **		0.583 **	0.31 9**		−0.406 ***	−0.203 ***	−0.354 **
		(13.689)	(3.163)		(13.159)	(2.588)		(−9.774)	(−4.036)	(−3.142)
Laziness attribution									−0.169 **	−0.126 *
									(−2.730)	(−2.062)
Responsibility-avoidance attribution									−0.189 ***	−0.172 **
									(−3.349)	(−3.119)
Human–AI task interdependence			0.079 *			0.022 *				0.427 **
			(1.995)			(2.150)				(3.252)
Perceived employee–AI collaboration × Human–AI task interdependence			0.039 **			0.065 **				0.069 **
			(2.638)			(2.681)				(2.883)
Constant	3.820 **	1.846 **	1.624 **	3.973 **	1.884 **	1.918 **	1.091 **	2.547 ***	3.215 **	4.192 **
	(12.491)	(6.414)	(3.727)	(11.954)	(5.945)	(3.968)	(3.779)	(8.563)	(10.696)	(9.529)
R^2^	0.143	0.433	0.471	0.084	0.378	0.412	0.392	0.517	0.568	0.595
adj. R^2^	0.129	0.422	0.456	0.069	0.366	0.395	0.382	0.508	0.557	0.581
F	10.215 ***	39.958 ***	36.080 ***	5.639 ***	31.832 ***	28.377 ***	39.518 ***	56.223 ***	53.282 ***	48.391 ***

*Note*. *** *p* < 0.001, ** *p* < 0.01, and * *p* < 0.05.

**Table 7:** Test of moderated mediating effects.

Mediating Variables	Moderator	Effect	BootSE	Boot LLCI	Boot ULCI
Laziness attribution	M − SD	−0.054	0.027	−0.109	0.003
	M	−0.060	0.029	−0.118	−0.011
	M + SD	−0.065	0.032	−0.130	−0.015
Responsibility-avoidance attribution	M − SD	−0.076	0.025	−0.130	−0.030
	M	−0.089	0.028	−0.146	−0.036
	M + SD	−0.101	0.034	−0.171	−0.039

*Note*. If the confidence interval (CI) does not include 0, the mediating effect is significant; otherwise, it is not significant.

**Table 8:** Descriptive statistics and correlation coefficients (experiment).

Variables	Mean	SD	1	2	3	4	5
Perceived employee–AI collaboration	0.500	0.501	1				
Human–AI task interdependence	0.500	0.501	−0.012	1			
Laziness attribution	2.804	1.432	0.436 **	0.407 **	1		
Responsibility-avoidance attribution	2.238	1.293	0.338 **	0.365 **	0.663 **	1	
Coworker helping behaviour	3.227	1.248	−0.418 **	−0.303 **	−0.489 **	−0.492 **	1

*Note*. For perceived employee–AI collaboration, 0 = low employee–AI collaboration, and 1 = high employee–AI collaboration. For human–AI task interdependence, 0 = low human–AI task interdependence, 1 = high human–AI task interdependence, ** *p* < 0.01.

**Table 9:** Regression results for the predictors of attribution mechanism and coworker helping behaviour (Experiment).

Variables	Laziness Attribution			Responsibility-Avoidance Attribution			Helping Behaviour			Helping Behaviour		
	Model 1			Model 2			Model 3			Model 4		
	b	SE	t	b	SE	t	b	SE	t	b	SE	t
Employee–AI collaboration	1.005 **	0.181	5.55	0.445 **	0.173	2.565	−0.504 **	0.158	−3.187	−0.323 *	0.159	−2.026
Human–AI task interdependence (TI)	0.942 **	0.181	5.207	0.533 **	0.173	3.078	−0.270	0.158	−1.710	−0.077	0.159	−0.487
AI × TI	0.486 **	0.168	2.894	0.839 *	0.245	3.417	−0.990 *	0.224	−4.415	−0.777 ***	0.219	−3.539
Laziness attribution										−0.091 **	0.034	−2.654
Responsibility-avoidance attribution										−0.201 *	0.058	−3.477
Constant	3.127 ***	0.484	6.457	3.116 ***	0.463	6.725	2.148 **	2.423	5.706	3.060 **	0.443	6.911
R^2^	0.389			0.313			0.386			0.437		
F	21.893 ***			15.721 ***			21.695 ***			21.719 ***		

*Note*. The *b* values are non-standardised regression coefficients. *** *p* < 0.001, ** *p* < 0.01, and * *p* < 0.05 (two-tailed test).

**Table 10:** Bootstrap test of the mediating effect (experiment).

Effects	Effect Size	95% LLCI	95% ULCI
Total effect	−0.984	0.123	−1.225
Total indirect effect	−0.412	0.087	−0.598
Perceived employees–AI collaboration → laziness attribution → helping behaviour	−0.187	0.081	−0.365
Perceived employee–AI collaboration → responsibility-avoidance attribution → helping behaviour	−0.225	0.071	−0.370

**Table 11:** Test of moderated mediating effect (experiment).

Mediating Variables	Moderating Variables	Effect Size	Standard Error (Boot SE)	95% LLCI	95% ULCI
Laziness attribution	Low (−1 SD)	−0.092	0.065	−0.224	0.029
	Mean	−0.114	0.080	−0.280	0.036
	High (+1 SD)	−0.136	0.098	−0.345	−0.041
Responsibility-avoidance attribution	Low (−1 SD)	−0.129	0.040	−0.177	−0.021
	Mean	−0.174	0.061	−0.299	−0.056
	High (+1 SD)	−0.258	0.095	−0.452	−0.080

*Note*. The indirect effect is the product of non-standardised coefficients, and the confidence intervals are based on 5000 bootstrap samples. The indirect effect was significant when the 95% CI did not include 0.

## Data Availability

Datasets are available upon request from the authors.

## References

[B1-behavsci-16-00985] Aiken L. S., West S. G. (1991). Multiple regression: Testing and interpreting interactions.

[B2-behavsci-16-00985] Asana (2024). State of AI at work.

[B3-behavsci-16-00985] Bai J. Y., Huan T. C., Leong A. M. W., Luo J. M., Fan D. X. F. (2025). Examining the influence of AI event strength on employee performance outcomes: Roles of AI rumination, AI-supported autonomy, and felt obligation for constructive change. International Journal of Hospitality Management.

[B4-behavsci-16-00985] Birkstedt T., Minkkinen M., Tandon A., Mäntymäki M. (2023). AI governance: Themes, knowledge gaps and future agendas. Internet Research.

[B5-behavsci-16-00985] Bockstedt J. C., Buckman J. R. (2026). Humans’ use of AI assistance: The effect of loss aversion on willingness to delegate decisions. Management Science.

[B6-behavsci-16-00985] Cao L., Chen C., Dong X., Wang M., Qin X. (2023). The dark side of AI identity: Investigating when and why AI identity entitles unethical behaviour. Computers in Human Behaviour.

[B7-behavsci-16-00985] Carson J., Mackey J., Alexander K., McAllister C., Phillipich M. (2024). Within-and between-person effects of causal attributions on relationship improvement following perceived incivility. Journal of Occupational and Organisational Psychology.

[B8-behavsci-16-00985] Castelo N., Bos M. W., Lehmann D. R. (2019). Task-dependent algorithm aversion. Journal of Marketing Research.

[B9-behavsci-16-00985] Choi J. Y., Choi S. Y., Shin N., Sun J. (2025). The impact of coworker justice on interpersonal behaviour: Do different types of trust make a difference?. Organization Management Journal.

[B10-behavsci-16-00985] Choudhary V., Marchetti A., Shrestha Y. R., Puranam P. (2025). Human-AI ensembles: When can they work?. Journal of Management.

[B11-behavsci-16-00985] Chowdhury S., Dey P., Joel-Edgar S., Bhattacharya S., Rodriguez-Espindola O., Abadie A., Truong L. (2023). Unlocking the value of artificial intelligence in human resource management through AI capability framework. Human Resource Management Review.

[B12-behavsci-16-00985] Chugh D., Kern M. C., Zhu Z., Lee S. (2014). Withstanding moral disengagement: Attachment security as an ethical intervention. Journal of Experimental Social Psychology.

[B13-behavsci-16-00985] Claessens S., Veitch P., Everett J. A. (2025). Negative perceptions of outsourcing to artificial intelligence. Computers in Human Behaviour.

[B14-behavsci-16-00985] David E. M., Kim T. Y., Rodgers M., Chen T. (2021). Helping while competing? The complex effects of competitive climates on the prosocial identity and performance relationship. Journal of Management Studies.

[B15-behavsci-16-00985] Doshi A. R., Hauser O. P. (2024). Generative AI enhances individual creativity but reduces the collective diversity of novel content. Science Advances.

[B16-behavsci-16-00985] Earp B. D., Porsdam Mann S., Liu P., Hannikainen I., Khan M. A., Chu Y., Savulescu J. (2024). Credit and blame for AI–generated content: Effects of personalisation in four countries. Annals of the New York Academy of Sciences.

[B17-behavsci-16-00985] Flavián C., Pérez-Rueda A., Belanche D., Casaló L. V. (2021). Intention to use analytical artificial intelligence (AI) in services—The effect of technology readiness and awareness. Journal of Service Management.

[B18-behavsci-16-00985] Fornell C., Larcker D. F. (1981). Evaluating structural equation models with unobservable variables and measurement error. Journal of Marketing Research.

[B19-behavsci-16-00985] Fügener A., Walzner D. D., Gupta A. (2026). Roles of artificial intelligence in collaboration with humans: Automation, augmentation, and the future of work. Management Science.

[B20-behavsci-16-00985] Grabner I., Klein A., Speckbacher G. (2022). Managing the trade-off between autonomy and task interdependence in creative teams: The role of organisational-level cultural control. Accounting, Organizations and Society.

[B21-behavsci-16-00985] Grote G., Parker S. K., Crowston K. (2026). Taming artificial intelligence: A theory of control-accountability alignment among AI developers and users. Academy of Management Review.

[B22-behavsci-16-00985] Hagtvedt L. P. (2025). Bright and dark imagining: How creators navigate moral consequences of developing ideas for artificial intelligence. Academy of Management Journal.

[B23-behavsci-16-00985] Hai S., Long T., Honora A., Japutra A., Guo T. (2025). The dark side of employee-generative AI collaboration in the workplace: An investigation on work alienation and employee expediency. International Journal of Information Management.

[B24-behavsci-16-00985] Halbesleben J. R., Wheeler A. R. (2015). To invest or not? The role of coworker support and trust in daily reciprocal gain spirals of helping behaviour. Journal of Management.

[B25-behavsci-16-00985] Hargie O., Stapleton K., Tourish D. (2010). Interpretations of CEO public apologies for the banking crisis: Attributions of blame and avoidance of responsibility. Organisation.

[B26-behavsci-16-00985] Harvey P., Madison K., Martinko M., Crook T. R., Crook T. A. (2014). Attribution theory in the organisational sciences: The road travelled and the path ahead. Academy of Management Perspectives.

[B27-behavsci-16-00985] Heider F. (1958). The psychology of interpersonal relations.

[B28-behavsci-16-00985] Hohenstein J., Jung M. (2020). AI as a moral crumple zone: The effects of AI-mediated communication on attribution and trust. Computers in Human Behaviour.

[B29-behavsci-16-00985] Hu W., Yu Z., Xu J., Lin W., Dang J., van Dijk E. (2026). Who is responsible for self-AI or others-AI collaboration? The effect of power and task outcome in responsibility attribution. Acta Psychologica.

[B30-behavsci-16-00985] Huang Y., Gursoy D. (2024). How does AI technology integration affect employees’ proactive service behaviors? A transactional theory of stress perspective. Journal of Retailing and Consumer Services.

[B31-behavsci-16-00985] Jia H., Zhong R., Xie X. (2021). Helping others makes me fit better: Effects of helping behaviour by newcomers and coworker-attributed motives on newcomers’ adjustment. Journal of Business and Psychology.

[B32-behavsci-16-00985] Jia N., Luo X., Fang Z., Liao C. (2024). When and how artificial intelligence augments employee creativity. Academy of Management Journal.

[B33-behavsci-16-00985] Jungherr A., Rauchfleisch A. (2025). Artificial Intelligence in deliberation: The AI penalty and the emergence of a new deliberative divide. Government Information Quarterly.

[B34-behavsci-16-00985] Kırcaburun K., Özdemir P. (2026). Moral disengagement and unethical generative AI use as the chain mediators between antagonistic personality and problematic generative AI use. Behavioral Sciences.

[B35-behavsci-16-00985] Kong H., Yin Z., Baruch Y., Yuan Y. (2023). The impact of trust in AI on career sustainability: The role of employee–AI collaboration and protean career orientation. Journal of Vocational Behaviour.

[B36-behavsci-16-00985] Leszczyński G., Gaczek P., Ławrynowicz M. (2026). The bright side of AI in hiring: Collaborating with algorithms supports ethical decision-making. Journal of Business Research.

[B37-behavsci-16-00985] Liden R. C., Wayne S. J., Bradway L. K. (1997). Task interdependence as a moderator of the relation between group control and performance. Human Relations.

[B38-behavsci-16-00985] Lindell M. K., Whitney D. J. (2001). Accounting for common method variance in cross-sectional research designs. Journal of Applied Psychology.

[B39-behavsci-16-00985] Malle B. F. (2006). The actor-observer asymmetry in attribution: A (surprising) meta-analysis. Psychological Bulletin.

[B40-behavsci-16-00985] Man Tang P., Koopman J., McClean S. T., Zhang J. H., Li C. H., De Cremer D., Lu Y., Ng C. T. S. (2022). When conscientious employees meet intelligent machines: An integrative approach inspired by complementarity theory and role theory. Academy of Management Journal.

[B41-behavsci-16-00985] McNealis R. (2026). Shame in the machine: Affective accountability and the ethics of AI. AI & Society.

[B42-behavsci-16-00985] Podsakoff N. P., Whiting S. W., Podsakoff P. M., Blume B. D. (2009). Individual-and organisational-level consequences of organisational citizenship behaviours: A meta-analysis. Journal of Applied Psychology.

[B43-behavsci-16-00985] Raisch S., Krakowski S. (2021). Artificial intelligence and management: The automation-augmentation paradox. Academy of Management Review.

[B44-behavsci-16-00985] Raj M., Berg J. M., Seamans R. (2026). The artificial intelligence disclosure penalty: Humans persistently devalue AI-generated creative writing. Journal of Experimental Psychology: General.

[B45-behavsci-16-00985] Reif J. A., Larrick R. P., Soll J. B. (2025). Evidence of a social evaluation penalty for using AI. Proceedings of the National Academy of Sciences of the United States of America.

[B46-behavsci-16-00985] Rodell J. B., Lynch J. W. (2016). Perceptions of employee volunteering: Is it “credited” or “stigmatised” by colleagues?. Academy of Management Journal.

[B47-behavsci-16-00985] Sahebi S., Formosa P., Bankins S. (2026). The AI penalty and disclosure paradox: Trust, authenticity and knowledge uptake in AI-mediated communication. Computers in Human Behavior: Artificial Humans.

[B48-behavsci-16-00985] Schilke O., Reimann M. (2025). The transparency dilemma: How AI disclosure erodes trust. Organisational Behaviour and Human Decision Processes.

[B49-behavsci-16-00985] Senoner J., Schallmoser S., Kratzwald B., Feuerriegel S., Netland T. (2024). Explainable AI improves task performance in human–AI collaboration. Scientific Reports.

[B50-behavsci-16-00985] Shaikh F., Afshan G., Anwar R. S., Abbas Z., Chana K. A. (2023). Analysing the impact of artificial intelligence on employee productivity: The mediating effect of knowledge sharing and well-being. Asia Pacific Journal of Human Resources.

[B51-behavsci-16-00985] Shrestha Y. R., Ben-Menahem S. M., Von Krogh G. (2019). Organisational decision-making structures in the age of artificial intelligence. California Management Review.

[B52-behavsci-16-00985] Stelmaszak M., Möhlmann M., Sørensen C. (2025). When algorithms delegate to humans: Exploring human-algorithm interaction at Uber. MIS Quarterly.

[B53-behavsci-16-00985] Tan L., Li J. (2025). Working with robots makes service employees counterproductive? The role of moral disengagement and task interdependence. Tourism Management.

[B54-behavsci-16-00985] Tang P. M., Koopman J., Mai K. M., De Cremer D., Zhang J. H., Reynders P., Ng C., Chen I. H. (2023). No person is an island: Unpacking the work and after-work consequences of interacting with artificial intelligence. Journal of Applied Psychology.

[B55-behavsci-16-00985] Teng R., Zhou S., Zheng W., Ma C. (2023). Artificial intelligence (AI) awareness and work withdrawal: Evaluating chained mediation through negative work-related rumination and emotional exhaustion. International Journal of Contemporary Hospitality Management.

[B56-behavsci-16-00985] Tissot T. T., Roth L. H. (2026). Anti-obesity medication use sparks effort-based sanctions and social penalties. Scientific Reports.

[B57-behavsci-16-00985] Tu W. (2024). Unpacking the accountability cube and its relationship with blame avoidance. Public Management Review.

[B58-behavsci-16-00985] Van der Vegt G. S., Janssen O. (2003). Joint impact of interdependence and group diversity on innovation. Journal of Management.

[B59-behavsci-16-00985] Van Dyne L., LePine J. A. (1998). Helping and voice extra-role behaviours: Evidence of construct and predictive validity. Academy of Management Journal.

[B60-behavsci-16-00985] Wang J., Xing Z., Zhang R. (2023). AI technology application and employee responsibility. Humanities and Social Sciences Communications.

[B61-behavsci-16-00985] Weiner B., Perry R. P., Magnusson J. (1988). An attributional analysis of reactions to stigmas. Journal of Personality and Social Psychology.

[B62-behavsci-16-00985] Woehr D. J., Arciniega L. M., González L., Stanley L. J. (2024). Live to work, work to live, and work as a necessary evil: An examination of the structure and stability of work ethic profiles. Group & Organization Management.

[B63-behavsci-16-00985] Xiao Q., Yan J., Bamber G. J. (2023). How does AI-enabled HR analytics influence employee resilience: Job crafting as a mediator and HRM system strength as a moderator. Personnel Review.

[B64-behavsci-16-00985] Xu C., Yao Z., Yi J. (2026). Put not down your hedge: Exploring the impact of coworkers’ AI usage on employees’ knowledge hiding. The Service Industries Journal.

[B65-behavsci-16-00985] Xu L., Tian H., Zhang Y., Yu F. (2026). Shifting accountability to artificial intelligence: Delegating challenging decisions to AI for responsibility avoidance. Journal of Business Research.

[B66-behavsci-16-00985] Zhao P., He G., Guan J. (2026). The ethical costs of artificial intelligence: Investigating how and when workplace artificial intelligence usage promotes employee unethical outcomes. Journal of Business Ethics.

[B67-behavsci-16-00985] Zhou X., Chen C., Li W., Yao Y., Cai F., Xu J., Qin X. (2025). How do coworkers interpret employee AI usage: Coworkers’ perceived morality and helping as responses to employee AI usage. Human Resource Management.

[B68-behavsci-16-00985] Zhu X., Zhang A., Zhang J. (2026). The double-edged sword of AI efficiency: Self-efficacy erosion as a mediator linking instant gratification and perceived AI efficacy to AI dependency. Behavioral Sciences.

